# Atlanta Medical College

**Published:** 1858-10

**Authors:** 


### EDITORIAL AND MISCELLANEOUS.

### ATLANTA MEDICAL COLLEGE.

The closing exercises of the last session in the Atlanta Medical College occurred on the 2d of September, upon which occasion the degree of M. D. was conferred upon 39 young gentlemen.

The Annual Address to the graduating class was delivered by Custis B. Nottingham, M. D., of Macon, Georgia, a gentlemen of distinction in the profession—the address is published in this number of onr Journal, and will be found an exceedingly elegant and chaste production, in reference to which, it may be most appropriately said, the "words are fitly spoken," like "apples of gold in pictures of silver."

From all that we can learn of his own "walk and conversation," he is the embodiment of what is presented in his address as the type of the true physician, he who appreciates and is faithful to the high responsibilities and sacred obligations of the Medical profession.

The Valedictory Address, upon the part of the class, was delivered by A. M. Moore of Georgia, and was a highly creditable production, not only to his head, but his heart; we have, indeed, rarely heard an address which was altogether so appropriate to the occasion.

From the graduating class we expect a great deal—we honestly believe that it is very rarely that a number of young men have left the College walls with a more decided and earnest purpose to be faithful to the obligations imposed by the assumption of so responsible a calling. Among them (we are greatly pleased to say it) were not only gentlemen of talent and cultivation, but those in whom were found eminent piety, the salutary and controlling influence of which was felt during their sojourn here, and will doubtless be made instrumental in the accomplishment of great good in their future career.

While it is of course a relief that the labors of the College have terminated, it is sincerely coupled with regret that the many pleasant associations connected with the relations of pupil and teacher have been sundered, but as it cannot otherwise be, in this world of ceaseless change, we can only say, go forth young gentlemen with stout hearts and honest purposes to the discharge of the practical duties of life, and may the richest blessings of Heaven attend you.

---

☞ The Annual Announcement of Lectures in the Atlanta Medical College, for the Session of 1859, with a Catalogue of Matriculates and Graduates of 1858, will be sent out with this number of the Journal.

By reference to this, it will be perceived, notwithstanding the terrible financial convulsion through which the country has passed in the last year, that there has been a decided increase in the number of Matriculates over last year, amounting in 1858 to 134, with 39 Graduates.

